# Microbial Interactions With Dissolved Organic Matter Drive Carbon Dynamics and Community Succession

**DOI:** 10.3389/fmicb.2018.01234

**Published:** 2018-06-08

**Authors:** Xiaoqin Wu, Liyou Wu, Yina Liu, Ping Zhang, Qinghao Li, Jizhong Zhou, Nancy J. Hess, Terry C. Hazen, Wanli Yang, Romy Chakraborty

**Affiliations:** ^1^Earth and Environmental Sciences, Lawrence Berkeley National Laboratory, Berkeley, CA, United States; ^2^Institute for Environmental Genomics, Department of Microbiology and Plant Biology, The University of Oklahoma, Norman, OK, United States; ^3^Environmental Molecular Sciences Laboratory, Earth and Biological Sciences Division, Pacific Northwest National Laboratory, Richland, WA, United States; ^4^Geochemical and Environmental Research Group, Texas A&M University, College Station, TX, United States; ^5^Advanced Light Source, Lawrence Berkeley National Laboratory, Berkeley, CA, United States; ^6^National Key Laboratory of Crystal Materials, School of Physics, Shandong University, Jinan, China; ^7^State Key Joint Laboratory of Environment Simulation and Pollution Control, School of Environment, Tsinghua University, Beijing, China; ^8^Department of Civil and Environmental Engineering, The University of Tennessee, Knoxville, Knoxville, TN, United States; ^9^Department of Microbiology, The University of Tennessee, Knoxville, Knoxville, TN, United States; ^10^Department of Earth and Planetary Sciences, The University of Tennessee, Knoxville, Knoxville, TN, United States; ^11^Institute for a Secure and Sustainable Environment, The University of Tennessee, Knoxville, Knoxville, TN, United States; ^12^Biosciences Division, Oak Ridge National Laboratory, Oak Ridge, TN, United States

**Keywords:** natural organic matter (NOM), molecular-level characterization, dynamic interactions, microbial succession, labile carbon, recalcitrant carbon, subsurface carbon cycling, microbe-carbon interactions

## Abstract

Knowledge of dynamic interactions between natural organic matter (NOM) and microbial communities is critical not only to delineate the routes of NOM degradation/transformation and carbon (C) fluxes, but also to understand microbial community evolution and succession in ecosystems. Yet, these processes in subsurface environments are usually studied independently, and a comprehensive view has been elusive thus far. In this study, we fed sediment-derived dissolved organic matter (DOM) to groundwater microbes and continually analyzed microbial transformation of DOM over a 50-day incubation. To document fine-scale changes in DOM chemistry, we applied high-resolution Fourier transform ion cyclotron resonance mass spectrometry (FT-ICR MS) and soft X-ray absorption spectroscopy (sXAS). We also monitored the trajectory of microbial biomass, community structure and activity over this time period. Together, these analyses provided an unprecedented comprehensive view of interactions between sediment-derived DOM and indigenous subsurface groundwater microbes. Microbial decomposition of labile C in DOM was immediately evident from biomass increase and total organic carbon (TOC) decrease. The change of microbial composition was closely related to DOM turnover: microbial community in early stages of incubation was influenced by relatively labile tannin- and protein-like compounds; while in later stages the community composition evolved to be most correlated with less labile lipid- and lignin-like compounds. These changes in microbial community structure and function, coupled with the contribution of microbial products to DOM pool affected the further transformation of DOM, culminating in stark changes to DOM composition over time. Our study demonstrates a distinct response of microbial communities to biotransformation of DOM, which improves our understanding of coupled interactions between sediment-derived DOM, microbial processes, and community structure in subsurface groundwater.

## Introduction

Natural organic matter (NOM) is the largest reactive reservoir of reduced carbon (C) on Earth (Bianchi, 2011), and transformation/decomposition of NOM is of fundamental interest due to its contributions to the global C cycle and C flux in ecosystems (Schlesinger and Andrews, 2000; Davidson and Janssens, 2006; Heimann and Reichstein, 2008). Until recently, it was widely accepted that soil NOM persisted as chemically stable ‘humic substances,’ complex mixtures of high molecular weight biopolymers and their degradation products, and was unlikely to be substantially degraded by microbes (Kelleher and Simpson, 2006). Recent insights indicated that the persistence of NOM is not just dependent on its intrinsic molecular structure, but also on other factors such as NOM concentration (Arrieta et al., 2015) and biophysico-chemical influences (e.g., microbial activity) drawn from the surrounding environment (Kleber, 2010; Schmidt et al., 2011; Kallenbach et al., 2016).

Microorganisms are key mediators in the formation, mobilization, transformation, and storage of NOM in various environments such as soil, sediment, marine, and freshwater (Carlson et al., 2004; Young et al., 2004; Jiao et al., 2010; Lin et al., 2014; Neumann et al., 2014; Xue et al., 2016). NOM chemistry affects microbial community structure and metabolic potential, as recently elucidated in marine (McCarren et al., 2010), soil (Ding et al., 2015), and groundwater environments (Zhang et al., 2015b). Concurrently, microbial-derived products and residues such as polysaccharides, proteins, cell wall polymers, along with a number of uncharacterized molecules also become integral components of NOM during those processes (Kogel-Knabner, 2002; Kelleher and Simpson, 2006; Koch et al., 2014; Osterholz et al., 2015; Kallenbach et al., 2016). Therefore, microbial community composition, ability of microbes to metabolize/transform NOM, and bioavailability of C substrates in NOM are intimately connected, but such interactions are not well documented in the environment.

In recent years, researchers have applied state-of-the-art instruments to investigate correlations between NOM chemistry and microbial populations (Oni et al., 2015). Identifying the molecular signatures of NOM is vital to understanding its biotransformation by microbes. Fourier transform ion cyclotron resonance mass spectrometry (FT-ICR MS) holds great promise for being able to provide both qualitative and quantitative description of NOM at molecular scale, and has been increasingly utilized over the past decade as a powerful approach toward characterizing NOM in environmental samples (Mann et al., 2015; Tfaily et al., 2015; Stegen et al., 2016; Linkhorst et al., 2017). Soft X-ray absorption spectroscopy (sXAS) can also provide fine-scale analysis of NOM due to its sensitivity to specific chemical states of C associated with different functional groups. This technique has been successfully applied to characterize soil extracts from different forest sites (Lehmann et al., 2008).

Previously, NOM was mostly extracted by acid/alkali treatment methods to define composition and identify functional groups. However, these harsh treatments also fundamentally change the native molecular structure of NOM, and the information gleaned is therefore not relevant or useful for NOM-microbe interactions. Instead, water-extractable NOM, i.e., dissolved organic matter (DOM), represents a more natural suite of organic molecules that microbes experience in their native environments, and thus is more relevant to microbes in sediment and soil despite being a smaller fraction of the whole (Guigue et al., 2015).

Dissolved organic matter from sediment is one of main C inputs to groundwater (Aiken, 2002) and consistently contributes to dissolved organic C pool in groundwater despite seasonal shift of organic C content in groundwater (Awoyemi et al., 2014). Subsurface DOM from deep sediment is generally believed to be enriched in weathered C relative to soil (A and B horizons) due to fewer inputs of relatively fresh forms of C from plants, animals, and other organisms. Temporal investigations linking fine-scale DOM turnover to microbial community structure and functional gene changes in subsurface environments are rare. Therefore, the goal of our study was to understand the interactions between groundwater microbes and sediment-derived DOM. We hypothesize that microbes may continuously shape and transform these sediment-derived dissolved organic C pools in groundwater toward more recalcitrant DOM, and this change in DOM chemistry will in turn determine the structure and function of developing microbial community.

We proceeded by designing microcosm experiments using DOM extracted from sediments adjacent to groundwater as C source to groundwater microbes. Microcosm is commonly used as a proxy to understand key *in situ* processes (Osterholz et al., 2015). In this study, the initial microbial cell concentration and organic C content in microcosm were kept very close to that present in groundwater at our field site. We applied a combination of advanced analytical techniques to investigate the linkage between fine-scale changes of DOM and the resultant shifts in microbial biomass, community structure, and metabolic potential. Successful integration of refined molecular diagnostic tools is fundamental to this work and has allowed us to investigate biotransformation of specific groups of DOM by microbes, which is a key step forward toward ecosystem-level understanding of C cycling in subsurface environments.

## Materials and Methods

### Sediment Collection

Sediment sample was obtained from a borehole FW305, at Oak Ridge Reservation Field Research Center (ORR-FRC), Oak Ridge, TN, at the depth of 4.6–5 m below ground surface (at groundwater level). The borehole was drilled adjoining a groundwater well GW305 and advanced using a dual tube (DT22) direct-push Geoprobe drill rig. During dual tube sampling, one set of rods was driven into the ground as an outer casing which received the driving force from the hammer and provided a sealed casing through which undisturbed sediment samples were recovered using inner rods. Sediment samples were recovered using disposable thin-walled polyvinyl chloride (PVC) liners (152.4 cm length × 2.86 cm I.D.) attached to 3.18 cm-outside diameter inner rods.

### Extraction of DOM From Sediment Sample

The sediment sample was freeze-dried and then extracted using Milli-Q water (18.2 MΩ⋅cm, 0.22 μm membrane filtered) via rotary shaking (170 rpm) overnight at 35°C, followed by sonication for 2 h. The ratio of water and sediment was 4:1 (w/w). The extracts were then centrifuged at 6000 *g* for 20 min. The supernatant was decanted and filtered through polycarbonate filter (0.2 μm pore-sized, Whatman), followed by filter-sterilization with polyethersulfone (PES) filters (0.22 μm pore-sized, Corning). The filtrate containing sediment-derived DOM was freeze-dried and stored at -20°C until use.

### Incubation of Microbes With Sediment-Derived DOM

The culture medium was prepared by adding an aliquot of freeze-dried sediment extract to synthetic groundwater at 100 mg/L. Synthetic groundwater was prepared according to a previous study (Martinez et al., 2014), and the ingredients were as follows: FeSO_4_ (2 μM), MnCl_2_ (5 μM), Na_2_MoO_4_ (8 μM), MgSO_4_ (0.8 mM), NaNO_3_ (7.5 mM), KCl (0.4 mM), KNO_3_ (7.5 mM), CaCl_2_ (0.2 mM), NaH_2_PO_4_ (5 mM), and amended with vitamin mixtures (Balch et al., 1979) at 5 ml/L. The medium was then filter-sterilized (0.22 μm pore-sized, PES, Corning) to remove any undissolved particles. The final total organic carbon (TOC) and total inorganic carbon (TIC) content of the medium was 8.0 and 0.5 mg/L, respectively.

Groundwater (pH 6.32, TOC 3.9 mg/L, TIC 4.9 mg/L, temperature 18.9°C) was sampled from well GW305 at ORR-FRC and shipped immediately to the lab with ice packs. At the time of sampling, dissolved oxygen in groundwater was measured to be 2.31 mg/L, indicating the water could be considered oxic. The groundwater was centrifuged at 6000 *g* for 20 min to concentrate microbes to a final cell concentration of 3.2 × 10^6^ cells/ml, prior to use as microbial inoculum in microcosms.

Microcosms were set up in 50-ml glass serum bottles. All bottles were cleaned with soap, and then thoroughly rinsed with acetone, methanol, and Milli-Q water to remove residual C. Clean bottles were autoclaved before use. Each bottle included 18 ml of medium containing sediment-derived DOM and 2 ml of microbial inoculum. The number of cells in microcosms was 10^5^ cells/ml, which was within the range of cell counts typical in ORR-FRC groundwater fluctuating between 10^4^ and 10^6^ cells/ml. Samples were incubated aerobically at room temperature (20°C) with periodic shaking. Three replicate bottles were sacrificed at each time point (days 1.5, 8, 13, 30, and 50), for biological and chemical analyses. At each sampling, 10 μl of culture was removed for acridine orange direct count (AODC) of cells (Hazen et al., 2010), and the rest was centrifuged at 10,000 *g* to separate microbes from culture broth. The cell pellet was stored at -80°C for further DNA extraction; and the supernatant was filtered through a syringe filter (0.2 μm, 25 mm, PES, Thermo Scientific) prior to storing at 4°C (up to 1 week) for DOM characterization.

Four control groups (with three replicates for each group) were included in this study (Supplementary Table S1): the first control set used glucose (1 mM) instead of sediment-derived DOM as C source to the microbes; the second control set lacked C amendments (i.e., without sediment-derived DOM); the third set of controls was uninoculated in order to monitor any abiotic changes of DOM over the course of incubation; and the fourth set was just uninoculated synthetic groundwater, used to monitor potential C contamination during incubation. All control groups were sampled at the end of experiment (day 50).

### Characterization of C

Several analytical techniques were applied to characterize DOM prior to and following incubations. Functional groups in the original sediment-derived DOM were determined by Ultraviolet–visible spectroscopy (UV/Vis). sXAS and FT-ICR MS were applied to investigate fine-scale chemical changes of DOM in microcosms. C analysis (TIC/TOC) of sediment and water samples were measured by Vario Max Analyzer (Elementar, Germany) and TOC-5050A Total Organic Carbon Analyzer (Shimadzu, Japan), respectively.

### sXAS

An aliquot of 10 ml filtered supernatant was freeze-dried for sXAS characterization. The C*-K* edge sXAS was performed in the iRIXS endstation (previously SXF) at Beamline 8.0.1 of the Advanced Light Source at Lawrence Berkeley National Laboratory (Qiao et al., 2017). The beamline is equipped with a undulator and a spherical grating monochromator that produced linearly polarized soft X-ray with a resolving power up to 6000. The samples were cooled with liquid nitrogen and checked carefully to avoid irradiation effect on the samples. The linear polarization (E vector) of the incident beam is 45° to the sample surface. The XAS signal was collected in total electron yield (TEY) mode. TEY spectra were obtained by measuring the compensating current upon incident photon energy with a probe depth of about 10 nm. All spectra were normalized to the incident photon flux monitored by the photocurrent from a clean gold mesh upstream. Energy resolution of the sXAS spectra was better than 0.15 eV without considering core-hole lifetime broadening effect.

### FT-ICR MS

Molecular composition of DOM in microcosms was determined by a FT-ICR MS located at the Environmental Molecular Sciences Laboratory (EMSL) at Pacific Northwest National Laboratory. To minimize the ion suppression caused by inorganic salts on FT-ICR instrument, a pre-clean procedure using solid phase extraction (SPE) was applied, described in the Supplementary Materials. SPE extracted samples were directly infused to a 12 Tesla FT-ICR MS (Bruker daltonics Inc., Billerica, MA, United States) with an electrospray ionization source equipped with a fused silica tube (30 μm i.d.) through an Agilent 1200 series pump (Agilent Technologies).

The flow rate of Agilent 1200 series pump was 4.0 μL/min. Experimental conditions were as follows: needle voltage, +4.4 kV; Q1 set to 100 m/z; and the heated resistively coated glass capillary operated at 180°C. These were the optimal parameters established in earlier DOM characterization experiments (Tfaily et al., 2015). Ion accumulation time for these samples was 1 s. The spectra were collected in 4 MWord and the resolution at 400 m/z was >300,000. In total, 96 individual scans were averaged for each sample. After internal calibration, the mass accuracy was <0.3 ppm for singly charged ions across a broad m/z range (i.e., 200–1200 m/z). Peaks with signal-to-noise ratio >7 were picked and elemental formulae were subsequently assigned with an in-house software based on the Compound Identification Algorithm (CIA) described by Kujawinski and Behn (2006). To avoid compound assignment ambiguity, only compounds with <0.3 ppm assignment error are reported.

In this study, we only detected singly charged ions in FT-ICR MS analysis; as such, m/z is reflecting the monoisotopic masses of the compounds detected. Therefore, molecular weights of the detected compounds can be calculated by correcting the detected m/z to neutral mass. We did not detect any multiply charged masses in the samples, after careful spectra examinations.

### Microbial Analyses

Extraction of microbial DNA was performed using PowerMax Soil DNA Isolation Kit (MO BIO Laboratories, Inc., Carlsbad, CA, United States) following the manufacturer’s protocol, and quantified using the Qubit dsDNA HS Assay Kit (Life Technologies, Eugene, OR, United States) with a Qubit fluorometer (Invitrogen, Eugene, OR, United States). The extracted DNA was used for 16S rRNA gene amplicon sequencing, and for GeoChip functional gene analysis.

### 16S rRNA Gene Amplicon Sequencing

The V4 region of the 16S rRNA genes was sequenced with a phasing amplicon sequencing approach with a two-step PCR library preparation strategy. Briefly, the first-round PCR was carried out with the target-only primer pair 515F (5′-GTGCCAGCMGCCGCGGTAA-3′) and 806R (5′-GGACTACHVGGGTWTCTAAT-3′). In the second-round PCR, phasing primers with Illumina functionalities, spacers, as well as barcodes on the reverse primers were introduced. Sample libraries were generated from purified PCR products and pooled for sequencing. Detailed procedures of PCR amplification, purification, library preparation were reported previously (Wu et al., 2015).

The MiSeq 500 cycles kit was used for 2 × 250 bp paired-ends sequencing on MiSeq machine (Illumina, San Diego, CA, United States). Raw sequences with perfect matches to barcodes were sorted to sample libraries and were trimmed by BTRIM with a threshold of quality control (QC) higher than 20 over a 5 bp window size and a minimum length of 100 bp (Kong, 2011). Forward and reverse reads with at least a 20 bp overlap and lower than 5% mismatches were joined with FLASH (Magoc and Salzberg, 2011). After trimming of ambiguous bases (i.e., N), joined sequences with lengths between 240 and 260 bp were subjected to chimera removal by U-Chime (Edgar et al., 2011). Operational taxonomic unit (OTU) clustering was through UCLUST at 97% similarity level by a *de novo* picking method (Edgar, 2010), and taxonomic assignment was through RDP classifier (Wang et al., 2007) with a minimal 50% confidence estimate. The above steps were performed through the Galaxy pipeline^1^ (Wen et al., 2017).

### GeoChip Analysis

Extracted DNA was used for GeoChip analysis as reported previously (Zhang et al., 2015a). Briefly, DNA (15 ng) was amplified and fluorescently labeled by whole community genome amplification with a modified (Wu et al., 2006) TempliPhi Kit (GE Healthcare, Piscataway, NJ, United States). Amplified and labeled DNA (2 μg) was then hybridized with GeoChip 5.0.

The GeoChip 5.0 used in this study contains a total of 161,961 probes targeting 1,447 functional gene families, covering 366,891 coding sequences. Specifically, 25,234 probes (15.6%) targeted 135 genes involved in C cycling processes. At the taxonomic level, the probes may target 6465 bacterial strains, 282 archaeal strains, 1073 eukaryotic strains, 1364 bacteriophages, and uncultured/unidentified/environmental organisms (Zhang et al., 2015c). Signal intensities were background-subtracted, and only spots with signal-to-noise ratio >2 were considered as positive and used for further analysis.

### Data Analysis

To control variation resulting from an unequal number of sequences across samples, sequence resampling was performed for each sample. Sequence resampling was performed after OTU generation at a rarefication sequence level based on the sample with the fewest number of sequences. Sequences from each sample are randomly drawn from the original pool until the rarefication sequence level is achieved. Once a sequence is drawn, it is excluded from further rounds of selection to prevent repetition.

Processing of the large FT-ICR MS data set, microbial community analysis, and all statistical tests were performed in R. For FT-ICR MS data, the assigned compounds were visualized in a van Krevelen diagram. It is important to note that a limitation of van Krevelen diagram visualizations is that molecules with different molecular formulas will plot at the same point if their oxygen/carbon (O/C) and hydrogen/carbon (H/C) ratios coincide. O/C and H/C ratios visualized by the van Krevelen diagram highlighted possible reaction pathways that might alter the oxygen content and saturation of the compounds. Additionally, key biochemical compound classes appeared in distinct locations on the van Krevelen diagram (Kim et al., 2003). As such, biochemical classification of FT-ICR MS data based on van Krevelen diagram has been widely applied to estimate possible classes of chemicals (e.g., lignin, amino sugar, protein, lipid, carbohydrate, tannin, and condensed aromatics) present in NOM (Kim et al., 2003; Sleighter and Hatcher, 2007; Minor et al., 2014; D’Andrilli et al., 2015; Tfaily et al., 2015). The boundary limits in van Krevelen diagram and other data analysis details are provided in the Supplementary Materials.

The 16S rRNA gene amplicon sequence data were analyzed using Bray–Curtis based non-metric multidimensional scaling (NMDS) ordination to show the community similarity according to taxonomic characteristics. Significance test of two compared objectives was performed using *t*-test. The canonical correspondence analysis (CCA) was used to determine which biochemical compositions of DOM were strongly related to the overall changes in microbial community structure. All the above statistics were performed using the “VEGAN” package in R.

## Results

### Characterization of DOM Extracted From Sediment

Total organic carbon and TIC content in sediment sample was 0.071 and 0.011%, respectively. The extraction method allowed recovery of approximately 3% of TOC and 2% of TIC from the sediment. TOC and TIC content in freeze-dried extracted DOM material was 14.5 and 1.1%, respectively.

Specific UV absorbance at low wavelengths (e.g., 254 or 280 nm) is often correlated to aromaticity (Krasner et al., 1996). The extracted DOM showed no significant absorbance across this range in UV/Vis spectrum (data not presented), suggesting that low amounts of aromatic/unsaturated compounds existed in the sediment-derived DOM.

### Microbial Growth and Decomposition of Labile C

**FIGURE 1:**
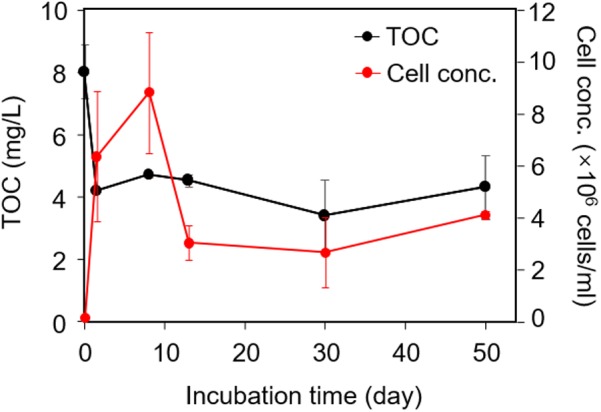
Changes in microbial biomass and total organic carbon (TOC) content in the microcosm-culture during the 50-day incubation. Microcosms included 18 ml of minimal medium containing sediment-derived dissolved organic matter (DOM) and 2 ml of microbial inoculum.

A rapid increase in microbial cell counts was observed in the initial phase of the experiment (**Figure 1**). The microbial cell counts decreased after peaking 9 × 10^6^ cells/ml at day 8 and stayed generally below 4 × 10^6^ cells/ml after day 13 (**Figure 1**). Concurrently, a sharp decrease in TOC in the culture was observed at an early stage, from 8.0 to 4.2 mg/L within 1.5 days (**Figure 1**), indicating a rapid utilization of labile C in sediment-derived DOM by microbes to support their fast growth. The TOC value remained at 3–5 mg/L until the end of the incubation after that. No significant decrease in TOC was observed in control group 3 (without inoculum) after a 50-day incubation (day 0: 8.0 ± 0.86 mg/L; day 50: 8.4 ± 0.78 mg/L), suggesting that abiotic decomposition of sediment-derived DOM and adsorption of DOM to the bottle were negligible under the experimental condition in this study. Also, TOC content in control group 4 was below detection limit at the beginning and at the end of experiment, suggesting that negligible C contamination (if any) from microcosm setup occurred during incubation.

### Changes in Functional Groups of DOM in Culture

**FIGURE 2:**
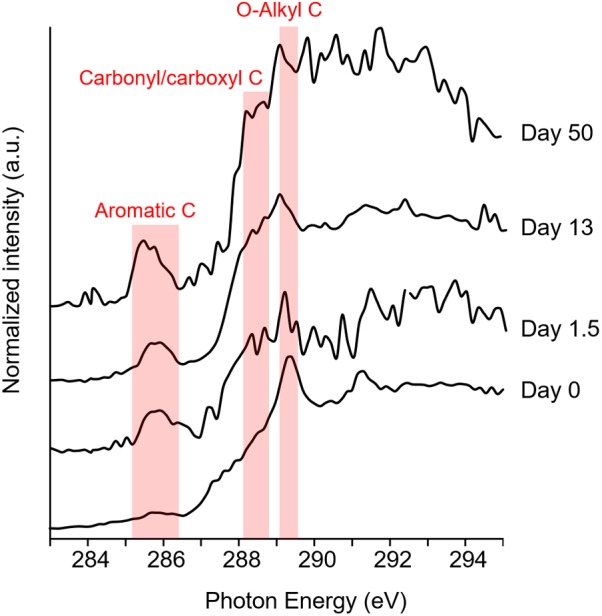
C *K*-edge sXAS spectra [in total electron yield (TEY) mode] of DOM during incubation (one of the three replicates was presented at each time point). C = C bond is shown as 285–287 eV; C = O bond is 288.2–288.7 eV; and C–O bond is 289.1–289.4 eV.

In C-*K* edge sXAS spectra, distinct spectral features and peak positions are characteristic of the coordination environment of C atoms and can provide detailed insights into the local chemistry (Solomon et al., 2009; Sedlmair et al., 2012). **Figure 2** shows directly the changes of sXAS lineshape upon incubation. The normalized intensity indicates the abundance of C bond in DOM material. At day 0, no significant aromatic C peak (π^∗^_C = C_, 285–287 eV) was observed, suggesting that compounds containing aryl functional group were present at very low amount in sediment-derived DOM, which agrees with the UV/Vis data. Within 1.5 days of incubation, spectral weight from C = C related absorption significantly increased (**Figure 2**) and remained high until the end of incubation, which can be attributed to microbially produced aromatics such as aromatic amino acids (e.g., phenylalanine, tyrosine, and tryptophan). Meanwhile, a gradual increase was observed in shoulder peaks between 288.2 and 288.7 eV from the C 1 s–π^∗^_C = O_ transition of carbonyl/carboxyl structures (**Figure 2**), attributable to metabolites such as nucleobases, lipids, and amino acids/peptides/proteins, which are often products of microbial metabolisms. Additionally, the distinct feature from O-alkyl C (σ^∗^_C-O_, 289.1–289.4 eV) of the day 0 sample diminished over time, and the overall lineshape at the high energies shows a broadening effect with many new peaks emerging (not noise as checked with multiple scans). This suggests continuous decomposition of original sediment-derived DOM and new C–O bond formation such as hydroxyl group connected to aliphatic C in polar metabolites of DOM. Therefore, these strong sXAS lineshape variations upon incubation clearly indicate the contribution of microbially derived products to DOM formation and genesis in culture.

### Changes in Molecular Composition of DOM in Culture

**FIGURE 3:**
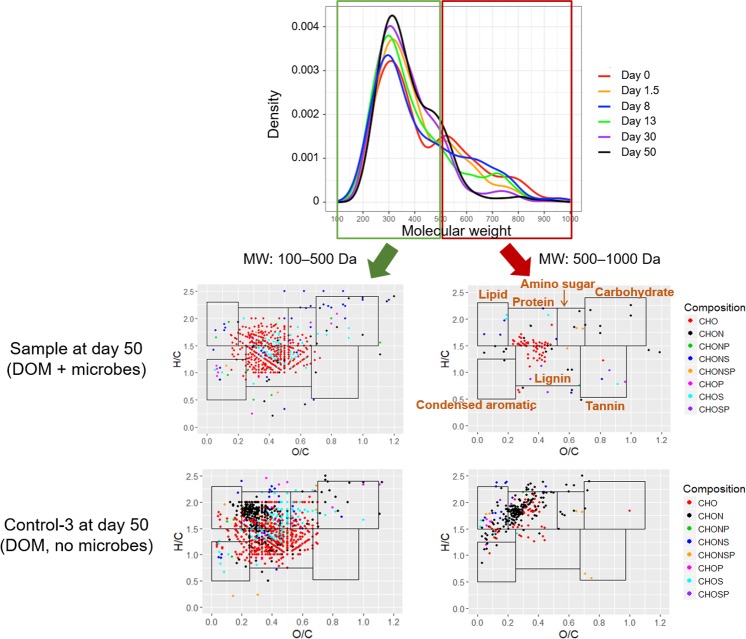
Distribution of molecular weights and van Krevelen diagram of compounds detected by Fourier transform ion cyclotron resonance mass spectrometry (FT-ICR MS) in DOM (one of the three replicates was presented). Boundary limits in van Krevelen diagram to constrain biochemical classifications were given in the Supplementary Materials.

Data from FT-ICR MS showed that a great part of detected molecules were compounds with molecular weight (MW) between 100 and 500 Da (**Figure 3**, here the FT-ICR MS features were considered only on presence/absence basis, not concentration), and their relative proportions (proportion of detected compounds) continuously increased during the 50-day incubation.

**FIGURE 4:**
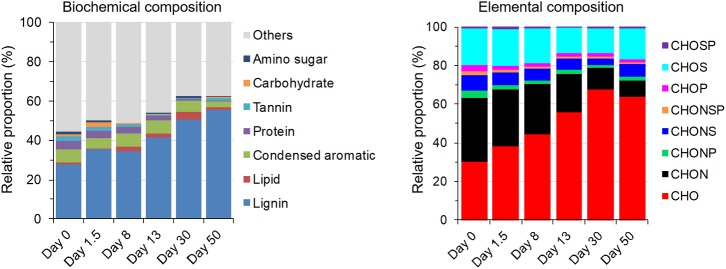
Biochemical and elemental composition of DOM measured by FT-ICR MS. Relative proportion was mean value of three replicates.

Meanwhile, relative proportions of large molecules (MW: 500–1000 Da) decreased over time (**Figure 3**). A comparison of van Krevelen diagrams of detected compounds in DOM with and without microbes at the end of incubation clearly showed a disappearance of protein-like compounds with elemental composition of C, H, O, and nitrogen (N) (CHON) (**Figure 3**), indicating utilization and/or degradation of these compounds by microbes. Accordingly, the relative proportions of protein-like and CHON compounds decreased during the 50-day incubation (**Figure 4**). On the other hand, relative proportions of lignin-like and CHO compounds continuously increased during incubation (**Figure 4**), probably because of their relatively resistances to biodegradation, contribution of microbial metabolites (control group 2, Supplementary Figure S1), and/or oxidation of certain DOM moieties (Fuchs et al., 2011).

### Shifts in Microbial Community Composition

A total of 402 bacterial OTUs were detected in this study. Phylogenetic classification demonstrated that community structure in microcosms was quite consistent over time at the phylum level but different at the order level. *Proteobacteria* was most abundant and dominant phylum (Supplementary Figure S2). At the order level, *Burkholderiales*, *Chitinophagales*, and *Rhodobacterales* were highly abundant in the original inoculum, and their relative abundances decreased during incubation; while relative abundance of some orders such as *Xanthomonadales*, *Sphingomonadales*, *Rhodospirillales*, *Rhizobiales*, and *Nitrosomonadales* increased over time (Supplementary Figure S3).

Non-metric multidimensional scaling ordination showed bacterial communities sampled at different time points differed significantly from each other (ANOSIM *R* = 0.77, *p* = 0.001) and shifted continuously (Supplementary Figure S4). At day 50, the community composition in experimental group was close to that in control group 2 (Supplementary Figure S4), suggesting that the property of DOM pool in these two groups might be similar. Community composition of control group 1 was very different from experimental group and control group 2 (Supplementary Figure S4), attributed to different C source in that group (glucose).

**FIGURE 5:**
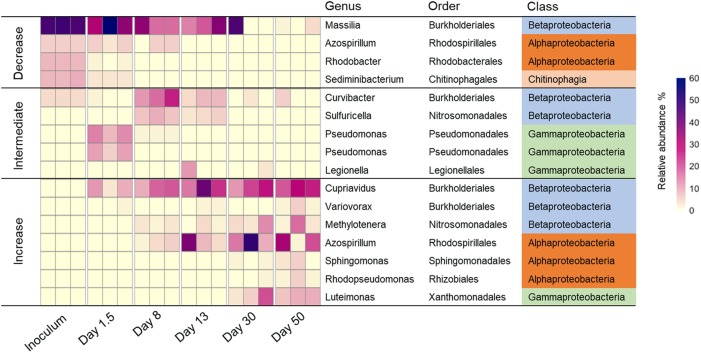
Relative abundance of major taxonomic species (>5% in any sample) across samples with three replicates at each time point. Here “increase” means the relative abundance of operational taxonomic unit (OTU) increased during incubation; “decrease” means the relative abundance decreased during incubation; and “intermediate” means the relative abundance increased in certain stages of incubation, then decreased.

The shift of dominant OTUs (>5% in any sample) showed a clear pattern of microbial succession (**Figure 5**). OTUs dominant in initial inoculum decreased significantly during incubation: relative abundance of *Massilia* sp. decreased from 46.2 ± 1.0 to 2.2 ± 2.1%; *Azospirillum* sp., *Rhodobacter* sp., and *Sediminibacterium* sp. decreased from 5–9% to below 0.05%. Meanwhile, some OTUs existing below 1% in initial inoculum increased in relative abundance during incubation, some OTUs just bloomed for a short intermediate period and then declined, e.g., *Pseudomonas* sp. and *Sulfuricella* sp., while some others persisted until the end of incubation, e.g., *Cupriavidus* sp. and *Azospirillum* sp. (**Figure 5**).

### Changes in Functional Genes Involved in C Degradation

**FIGURE 6:**
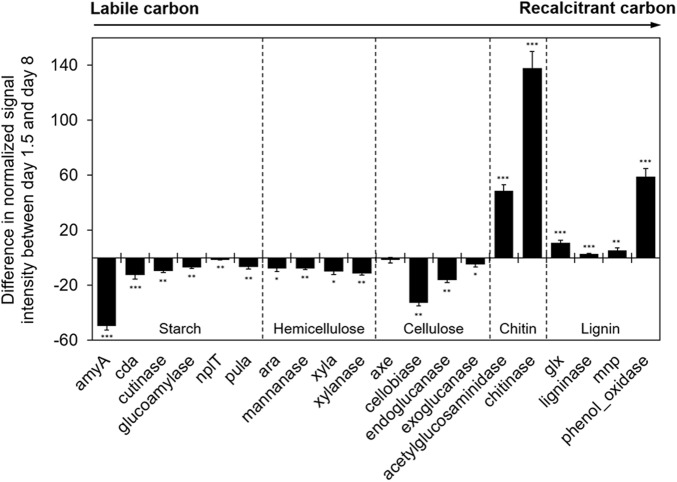
Difference in normalized signal intensity of detected functional genes involved in C degradation between days 1.5 and 8 from GeoChip data. The targeted substrates were arranged in order from labile to recalcitrant C. Significance is indicated by ^∗∗∗^ when *p* < 0.001, ^∗∗^ when *p* < 0.01, and ^∗^ when *p* < 0.05.

To obtain mechanistic insights into how DOM chemistry influences metabolic function of microbial communities, GeoChip data were examined by focusing on functional genes involved in degradation of labile to recalcitrant C substrates (He et al., 2010; Xue et al., 2016). Results of TOC analysis (**Figure 1**) indicated that labile C was quickly depleted under microbial activity after 1.5 days. This change of C pool resulted in corresponding shift in metabolic potential of the community. Similar to control group 1 which used labile C (glucose) as C source, intensities of fourteen detected genes involved in relatively labile C (starch, hemicellulose, and cellulose) degradation were high in early stages of incubation when labile C was still present in the culture (Supplementary Figure S5). After 1.5 days, labile C was depleted and thus the intensities of these genes decreased and stayed low in later stages (Supplementary Figure S5). Most of these genes (except *ax*) showed a significant (*p* < 0.05) decrease in abundance in samples at day 8 compared to those at day 1.5 (**Figure 6**), which was in accordance with chemical analysis results. On the other hand, the abundance of six detected genes involved in recalcitrant C (chitin and lignin) degradation increased significantly (*p* < 0.05) at day 8 compared to day 1.5 (**Figure 6**), suggesting that the metabolic function of microbial communities for degrading recalcitrant C was enhanced after labile C was consumed.

## Discussion

Although DOM drives microbial communities in natural ecosystems, little is known about the correlation between availability of naturally occurring DOM to microbes, microbe-catalyzed DOM transformations, and the resultant microbial community response in subsurface environments. In this study, we applied a combination of biophysico-chemical tools to document these changes in DOM chemistry and microbial communities.

In the earliest stage of incubation, microorganisms preferably utilized labile C in DOM, as indicated by the rapid increase in cell counts and corresponding decrease in TOC (**Figure 1**). Analysis of microbial community structure showed a rapid enrichment of *Pseudomonas* in early stage: relative abundances of two *Pseudomonas* spp. increased from 0.4–0.8% in inoculum to 5–14% at day 1.5 (**Figure 5**). *Pseudomonas* was often reported to be dominant genus in microcosms amended with labile C such as acetate (Cui et al., 2016) or glucose (Ghosh and Leff, 2013). Also, some species such as *Pseudomonas aeruginosa*, are known to be efficient competitors for resources through secretion of antibiotics (Hibbing et al., 2010) and toxins (Russell et al., 2011). It is therefore not surprising that *Pseudomonas* became abundant species in early stage of incubation when labile C was still available.

After 1.5 days, TOC content held constant, and microbial biomass increased slowly followed by a significant reduction after day 8 (**Figure 1**). This could indicate a change of microbial physiological state from activity and growth to maintenance, following a depletion of labile C in the culture. Corresponding to the decrease in bioavailable labile C, the apparent abundance of genes related to labile C degradation such as *amyA* (for degradation of starch) significantly decreased after day 1.5. Meanwhile, genes related to recalcitrant C degradation such as those encoding chitinase (for degradation of chitin) and phenol oxidases (for degradation of lignin) increased (**Figure 6**). *Pseudomonas* sp. exhibited a fast decline in relative abundance from 5–14% at day 1.5 to 0.2–2.2% at day 8 and stayed below 0.4% through the end of incubation (**Figure 5**), potentially indicating a response to the loss of labile C. Some genera such as *Curvibacter* and *Sulfuricella* were transiently dominant (from days 8 to 13) (**Figure 5**), and we speculate this might be directed by their ability to utilize specific types of C in DOM (Lau et al., 2016; Ma et al., 2016).

Some microbial species abundant in the inoculum, such as *Massilia* sp., *Azospirillum* sp., *Rhodobacter* sp., and *Sediminibacterium* sp., gradually declined during incubation (**Figure 5**). Since other physiological conditions such as pH or temperature remained the same throughout incubation (data not shown), we believed key causes for this decline included reduction in electron donor quantity and quality, or competition between microbes for C resources. As an example, *Massilia*, which are primarily rhizosphere and root colonizing bacteria (Green et al., 2007; Ofek et al., 2012), were considered as copiotrophs in rhizosphere ecology (Ofek et al., 2012), implying their preferential utilization of labile C. Also, *Massilia* were usually involved in early stages of bacterial succession in the rhizoplane, when C and energy sources were abundant, conceding to more competitive species as resources become limiting (Ofek et al., 2012), which was similar to what we observed in this study.

As relative proportion of recalcitrant C such as lignin-like compounds gradually became a greater proportion of DOM during incubation (**Figure 4**), a shift in microbial community structure was observed in response to this change of C pool. *Cupriavidus* sp., *Azospirillum* sp., and *Luteimonas* sp. gradually became dominant in later stages of incubation (**Figure 5**). A large number of genes expressed in these bacteria were linked to degradation of various types of compounds such as alicyclic compounds, proteinogenic amino acids, and recalcitrant aromatic compounds (Ghosal et al., 1985; Schlomann et al., 1990; Clement et al., 1995; Lykidis et al., 2010), suggesting that a community dominated by these bacteria probably harbored the metabolic potential of utilizing diverse C sources including recalcitrant C in DOM.

**FIGURE 7:**
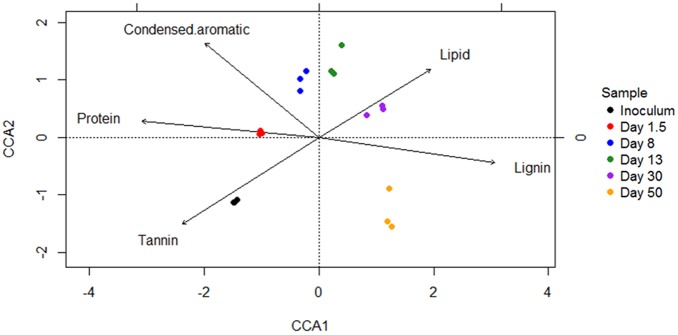
Canonical correspondence analysis (CCA) ordination plot shows significant (*p* = 0.001) correlations between microbial community structures (symbols) and biochemical variables (arrows). Arrows indicate the direction and magnitude of measurable variables associated with community structures.

Canonical correspondence analysis was performed to identify key biochemical compounds influencing microbial community structure. Shifts in microbial community composition were significantly (*p* = 0.001) correlated with changes in relative proportion of tannin-, protein-, condensed aromatic-, lipid-, and lignin-like compounds during incubation (**Figure 7**). Specifically, microbial community in early stages of incubation was influenced by relatively labile tannin- and protein-like compounds; while in later stages (e.g., days 30, 50), community composition evolved to be most correlated with less labile lipid- and lignin-like compounds.

The recalcitrant C pool was a result of depletion of sediment-derived labile C and addition of microbial-derived recalcitrant compounds and other uncharacterized compounds (Osterholz et al., 2015). We demonstrated the existence of microbial products in DOM pool, as indicated by aromatic C in sXAS spectra (**Figure 2**) and compounds detected by FT-ICR MS in control group 2 (Supplementary Figure S1). These microbial products likely included extracellular metabolites (Goodwin et al., 2015) and bacterial lysates released from dead cells (Turnbull et al., 2016). In this study, more than one third of detected microbial products were denoted as recalcitrant lignin-like compounds (Supplementary Figure S1).

Nitrogen (N) is also a key component of DOM (Wiegner and Seitzinger, 2004). Dissolved organic nitrogen (DON) is comprised of a continuum of compounds ranging from high-molecular weight polymers, e.g., polypeptides, to low-molecular weight monomers, e.g., amino acids and urea (Antia et al., 1991; Zehr and Ward, 2002), and therefore can provide both C and N for microbial communities (Antia et al., 1991; Zehr and Ward, 2002; Berman and Bronk, 2003; Ghosh and Leff, 2013). In this study, the relative proportion of compounds with CHON molecular formulae, which were mostly protein-like compounds (**Figure 4**), decreased gradually during incubation (**Figure 4**), indicating that these DON were likely continuously utilized by microorganisms when labile C was limited, serving as supplementary C and N sources.

In summary, our findings clearly demonstrated that microbial biomass, community structure, and microbial functions were closely related to the property and molecular composition of DOM. We were able to identify microbes that responded to and metabolized relatively labile or recalcitrant C, gain insights into microbial mechanisms (genes) employed for transforming DOM, and document fine-scale changes in DOM composition. A quick turnover and mineralization of DOM was observed due to the rapid degradation of labile C by indigenous microorganisms. As the C pool transitioned toward recalcitrant C, the dominant bacterial species shifted to the ones that might possess a greater diversity of C degradation potential. As a feedback mechanism, microbial metabolic activity affected the transformation/metabolism of specific types of compounds in DOM (e.g., proteins), and microbially produced compounds also contributed to the total DOM pool, resulting in dynamic changes in DOM property and composition. Our results demonstrated that C cycling in subsurface environments was orchestrated by temporal succession of microbes that utilized specific C pools in available DOM. In natural environments, such interactions between DOM and microorganisms dictate the ultimate fate of C.

## Sequence Information

The sequence information has been made available through the National Center for Biotechnology Information (NCBI). Accession: SRP139592.

## Author Contributions

XW and RC designed and managed the study. XW, LW, YL, PZ, and QL generated and analyzed the data. XW, JZ, NH, TH, WY, and RC prepared and wrote the manuscript.

## Conflict of Interest Statement

The authors declare that the research was conducted in the absence of any commercial or financial relationships that could be construed as a potential conflict of interest.

## Footnotes

1 http://zhoulab5.rccc.ou.edu:8080/root/login?redirect=%2F
