# Supplementary material for: Microbial Interactions With Dissolved Organic Matter Drive Carbon Dynamics and Community Succession
Source: Front Microbiol. 2018 Jun 8;9:1234. doi: 10.3389/fmicb.2018.01234 (PMC6002664; doi:10.3389/fmicb.2018.01234)
Supplement: Supplementary file 1 [file Data_Sheet_1.docx]

**Supplementary Material**

**Microbial interactions with dissolved organic matter drive carbon dynamics and community succession**

Xiaoqin Wu^1^, Liyou Wu^2^, Yina Liu^3,4^, Ping Zhang^2^, Qinghao Li^5,6^, Jizhong Zhou^1,2,7^, Nancy J. Hess^3^, Terry C. Hazen^8,9,10,11,12^, Wanli Yang^5^, Romy Chakraborty^1*^

^1^ Earth and Environmental Sciences, Lawrence Berkeley National Laboratory, Berkeley, California 94720, United States

^2^ Institute for Environmental Genomics, Department of Microbiology and Plant Biology, University of Oklahoma, Norman, Oklahoma 73019, United States

^3^ Environmental Molecular Sciences Laboratory, Earth and Biological Sciences Division, Pacific Northwest National Laboratory, Richland, Washington 99354, United States

^4^ Geochemical and Environmental Research Group, Texas A&M University, College Station, Texas 77845, United States

^5^ Advanced Light Source, Lawrence Berkeley National Laboratory, Berkeley, California 94720, United States

^6^ National Key Laboratory of Crystal Materials, School of Physics, Shandong University, Jinan, Shandong 250100, China

^7^ State Key Joint Laboratory of Environment Simulation and Pollution Control, School of Environment, Tsinghua University, Beijing, China

^8^ Department of Civil & Environmental Engineering, University of Tennessee, Knoxville, Tennessee 37996, United States

^9^ Department of Microbiology, University of Tennessee, Knoxville, Tennessee 37996, United States

^10^ Department of Earth & Planetary Sciences, University of Tennessee, Knoxville, Tennessee 37996, United States

^11^ Institute for Secure and Sustainable Environment, University of Tennessee, Knoxville, Tennessee 37996, United States

^12^ Biosciences Division, Oak Ridge National Laboratory, Oak Ridge, Tennessee 37831, United States

*Corresponding author: [rchakraborty@lbl.gov](mailto:rchakraborty@lbl.gov)

**Materials and Methods**

***Solid phase extraction (SPE) procedure***

Cartridges containing a styrene-divinylbenzene copolymer (PPL) sorbent was chosen for SPE based on previous studies (Dittmar *et al*., 2008). In this study, Bond Elut PPL cartridge (50 mg, 1 ml, Agilent Technologies) was used as SPE cartridge and was pre-conditioned with 1 ml of methanol followed by 5 ml of 0.01 M HCl. An aliquot of 5 ml acidified supernatant (pH = 2) was loaded on the pre-conditioned cartridge under gravity, during which organic compounds were retained on the cartridge while inorganic salts were passed through with the flow. The cartridge was washed with 5 ml of 0.01 M HCl and dried under N_2_. The organic compounds were eluted with 1 ml of methanol.

***Boundary limits in van Krevelen diagram to constrain the biochemical classifications***

The van Krevelen diagram provides a means to assign the compounds present in samples to major biochemical classes (e.g., lipid, protein, amino sugar, lignin, etc.) based on their H/C and O/C ratios of assigned molecular formula. The boundary limits in van Krevelen diagram were modified based on Minor *et al*. (2014) and Rivas-Ubach *et al*. (2018).

- Lipid: O/C >= 0 & O/C <= 0.2 & H/C >= 1.5 & H/C <= 2.3 & N/C <= 0.04 & P/C <= 0.03;
- Protein: O/C >= 0.2 & O/C <= 0.52 & H/C >= 1.5 & H/C <= 2.2 & N/C >= 0.178 & N/C <= 0.44 & P/C <= 0.06;
- Amino sugar: O/C >= 0.52 & O/C <= 0.7 & H/C >= 1.5 & H/C <= 2.2 & N/C > 0.07 & N/C <= 0.182 & P/C < 0.167;
- Carbohydrate: O/C >= 0.7 & O/C <= 1.1 & H/C >= 1.5 & H/C <= 2.4 & N = 0 & P = 0;
- Condensed aromatics: O/C >= 0 & O/C <= 0.25 & H/C >= 0.5 & H/C <= 1.25;
- Lignin: O/C >= 0.25 & O/C <= 0.67 & H/C >= 0.75 & H/C <= 1.5;
- Tannin: O/C >= 0.67 & O/C <= 0.97 & H/C >= 0.53 & H/C <= 1.5.

**Reference:**

Dittmar T, Koch B, Hertkorn N, Kattner G. (2008). A simple and efficient method for the solid-phase extraction of dissolved organic matter (SPE-DOM) from seawater. *Limnol Oceanogr-Methods,* **6**: 230–235.

Minor EC, Swenson MM, Mattson BM, Oyler AR. (2014). Structural characterization of dissolved organic matter: a review of current techniques for isolation and analysis. *Environ Sci Process Impacts*, **16**: 2064–2079.

Rivas-Ubach A, Liu Y, Bianchi T, Tolić N, Jansson C, Paša-Tolić L. (2018). Moving beyond the van Krevelen diagram: a new stoichiometric approach for compound classification in organisms. *Anal Chem*, 90: 6152–6160.

Table S1. Experimental design of the study. Synthetic groundwater was used as the basic medium for all groups. Three replicates were included for each group.

| **Group** | **Carbon source** | **Inoculum** |
| --- | --- | --- |
| Experimental group | Sediment-extracted organic matter | FRC groundwater microbes |
| Control group 1 | Glucose | FRC groundwater microbes |
| Control group 2 | N.A.^*^ | FRC groundwater microbes |
| Control group 3 | Sediment-extracted organic matter | N.A. |
| Control group 4 | N.A. | N.A. |

^*^ N.A.: not applicable.

Figure S1. Van Krevelen diagram and biochemical composition of detected compounds by FT-ICR MS in control group 2 at day 50. One of the three replicates was presented in van Krevelen diagram, and relative proportion was mean value of three replicates.

Figure S2. Relative abundance of each taxonomic phylum (> 1% in any sample). Control group 1 and 2 were sampled at day 50.


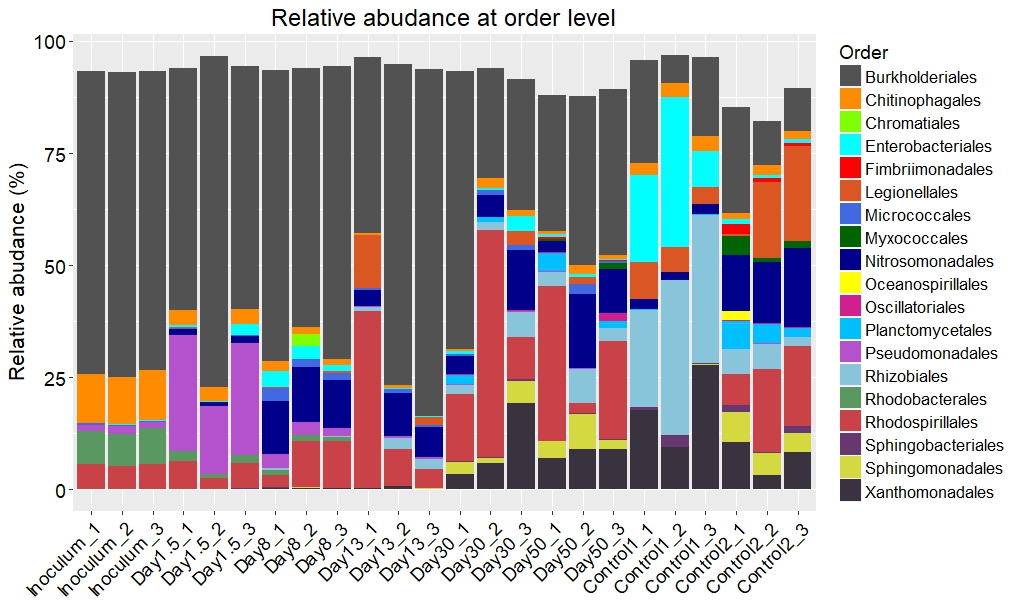


Figure S3. Relative abundance of each taxonomic order (> 1% in any sample). Control group 1 and 2 were sampled at day 50.

Figure S4. Non-metric multidimensional scaling (NMDS) based on Bray-Curtis dissimilarities of bacterial community composition. Control group 1 and 2 were sampled at day 50.

Figure S5. Normalized signal intensities of functional genes involved in degradation of labile C from GeoChip data. Control group 1 and 2 were sampled at day 50.
